# Assessing and understanding sedentary behaviour in office-based working adults: a mixed-method approach

**DOI:** 10.1186/s12889-016-3023-z

**Published:** 2016-04-27

**Authors:** Clarice N. Waters, Er Pei Ling, Anne H. Y. Chu, Sheryl H. X. Ng, Audrey Chia, Yee Wei Lim, Falk Müller-Riemenschneider

**Affiliations:** Saw Swee Hock School of Public Health, National University of Singapore, 12 Science Drive 2, Tahir Foundation Building, Singapore, 117549 Singapore; NUS Business School, National University of Singapore, 15 Kent Ridge Drive, Mochtar Riady Building, Singapore, 119245 Singapore; Institute for Social Medicine, Epidemiology and Health Economics, Charité University Medical Centre Berlin, Berlin, Germany

**Keywords:** Adult, Occupational Health, Sedentary lifestyle, Workplace

## Abstract

**Background:**

Sedentary behaviours (SB) can be characterized by low energy expenditure in a reclining position (e.g., sitting) often associated with work and transport. Prolonged SB is associated with increased risk for chronic conditions, and due to technological advances, the working population is in office settings with high occupational exposure to SB. This study aims to assess SB among office workers, as well as barriers and strategies towards reducing SB in the work setting.

**Methods:**

Using a mixed-methods approach guided by the socio-ecological framework, non-academic office workers from a professional school in a large public university were recruited. Of 180 eligible office workers, 40 enrolled and completed all assessments. Self-reported and objectively measured SB and activity levels were captured. Focus group discussion (FGD) were conducted to further understand perceptions, barriers, and strategies to reducing workplace SB. Environmental factors were systematically evaluated by trained research staff using an adapted version of the Checklist for Health Promotion Environments at Worksites (CHEW). Thematic analysis of FGD was conducted and descriptive analysis of quantitative data was performed.

**Results:**

The sample was mostly Chinese (*n* = 33, 80 %) with a total of 24 (60 %) female participants. Most participants worked five days a week for about 9.5(0.5) hrs/day. Accelerometer data show that participants spend the majority of their days in sedentary activities both on workdays (76.9 %) and non-workdays (69.5 %). Self-report data confirm these findings with median sitting time of 420(180) minutes at work. From qualitative analyses, major barriers to reducing SB emerged, including the following themes: workplace social and cultural norms, personal factors, job scope, and physical building/office infrastructure. CHEW results confirm a lack of support from the physical infrastructure and information environment to reducing SB.

**Conclusions:**

There is high SB among office workers in this sample. We identified multiple levels of influence for prolonged occupational SB, with a particular emphasis on workplace norms and infrastructure as important barriers to reducing SB and increasing PA. A larger, representative sample of the Singaporean population is needed to confirm our findings but it seems that any intervention aimed at reducing SB in the workplace should target individual, environmental, and organizational levels.

**Electronic supplementary material:**

The online version of this article (doi:10.1186/s12889-016-3023-z) contains supplementary material, which is available to authorized users.

## Background

Modernisation of societies has resulted in the high reliance of technology, especially in the workplace; and with this, comes the increasing trend of workplace sedentary behaviour (SB) [1]. SB is denoted by low levels of energy expenditure in a sitting or reclining posture, prevalent in the domains relating to occupation, transportation, and recreation/leisure activities [2–5]. Common SB may include excessive sitting related to television viewing, video game playing, computer or other screen device use, driving automobiles, and reading [2]. An individual can engage in excessive sitting while still meeting the recommendations of 150 min of physical activity (PA) per week in daily activities [6]. Therefore, it is important to note that SB is distinct from being physically inactive [4, 7, 8]. High SB engagement has been associated with an increased risk for all-cause mortality, higher body mass index (BMI), and an array of chronic conditions, while a reduction in excessive SB may have beneficial health impacts [1, 4, 7, 9–16]. With a large portion of today’s workforce in office-based settings and the large amount of daily waking hours spent at work, occupation-related SB is of critical public health concern [17].

Across 20 different countries, the median sitting time on a usual weekday was reported to be five hours per day [18]. Taiwan, Hong Kong, and Japan were among the sampled countries that reported higher median sitting times, close to six hours per day [18]. In other studies conducted in the United States (US), United Kingdom (UK), and Australia that used objective and self-report measurements of SB, adults may spend nearly nine hours per day sedentary [19–21]. These trends are similar in Singapore. It was found that about 37 % of Singaporean adults aged 18 to 79 years engage in excessive sitting for more than eight hours per day [22].

In examining domain-specific SB, studies have shown that on a typical working day an individual may spend more than half the day sedentary, in bouts of 30 min or more [20, 21, 23, 24]. While sitting in the workplace is heavily dependent on the occupation type and types of tasks involved, workforces of many industrialized nations are professional jobs in office-type settings [23]. In Singapore, approximately 80 % of the nation’s workforce is in professional occupations, presumably engaging in high levels of SB at work [25].

Specific to the work place domain, a variety of factors may influence SB, such as personal habits, social norms of sitting at a desk, or the availability of certain office furniture. This is in line with a socio-ecological model of health which conceptualizes multiple levels of influence, including intrapersonal-, interpersonal-, organizational-, environmental- and policy-related factors [7]. It is therefore necessary to have a comprehensive understanding of SB and relevant factors to appropriately establish behaviour change interventions [7]. Currently, research in the area of work-related SB is in a nascent stage with no standard in assessment of SB, as both self-report and objective assessment offer distinct information [5, 15]. Combining different methods of assessments may yield more in depth information on SB [15]. There appears to be a scarcity of published research aimed at combining different methodological assessments for a more comprehensive understanding of SB. Therefore, to address the current gaps in the literature, the objective of this study is to conduct a comprehensive workplace SB assessment. Specifically, this study aims to describe SB among Singaporean office-based workers. It further aims to identify individual, interpersonal, organizational and environmental barriers and potential strategies towards the reduction of SB among office workers in Singapore.

## Methods

### Approach

Under the socio-ecological framework, this cross-sectional study used a mixed-method approach in assessing different levels of influences on SB in the workplace. Both quantitative and qualitative data related to workplace SB were collected. This study was approved by the National University of Singapore Institutional Review Board (NUS IRB reference B14-060).

### Setting and sample

For this study, eligible participants were recruited from a professional school within a local flagship university if they met the following inclusion criteria: non-academic staff, office-based workers ≥21 years old, working ≥4 days per week, proficient in English, not physically disabled or handicapped, and not pregnant. Among 180 office-based employees of the professional school, all were invited to join the research study through multiple personal engagements at school-level meetings, senior management announcements, and department-wide mass emails. During recruitment sessions, interested and eligible participants were enrolled after signing the informed consent. All assessment measures were collected from July to September 2014. Anthropometric measurements were taken and accelerometers were distributed with instructions for wear and return. Over the course of three months, a total of 10 recruitment and enrolment sessions were held. Out of 180 office staff, 43 were enrolled as participants and 40 completed all assessment measures. The worksite for this particular professional school spans across two separate, but inter-connected buildings.

### Measures

#### Quantitative

A series of quantitative measures via self-report questionnaires and objective measures were used to capture SB among participants. An online survey was developed using Qualtrics 2014 comprising of previously used questionnaires on sociodemographic characteristics, overall and domain-specific SB on working and non-working days, PA, current health status and lifestyle behaviours [6, 26, 27]. For details of the survey, refer to Additional file 1.

Anthropometric measurements were taken by two trained research staff using a digital column weighing scale (Seca 769) with measuring rod (Seca 220) attached. Weight was measured to the nearest 0.1 kg without objects in pockets or heavy jackets. Height was measured nearest 0.1 cm without shoes and the participant was looking straight forward. Waist and hip circumferences were measured using a flexible measuring tape to nearest 1 cm. All anthropometric measurements were taken twice to ensure accuracy. Body mass index (BMI) and waist-hip ratio (WHR) were subsequently calculated. BMI was categorized based on the cut off points for Asian populations: 18.5–22.9 kg/m^2^ as “normal”, 23–27.4 kg/m^2^ as “overweight”, and ≥27.5 kg/m^2^ as “obese” [28, 29].

Actigraph GT3X + BT accelerometers were worn for one week to objectively capture SB and PA. The device was placed over the right hip and attached by an elastic band to secure around the waist. In addition, participants were given an accelerometer time sheet to indicate their transportation time to travel from places, working hours on working days, time spent for any structured exercise, duration of time and reasons for not wearing the accelerometer based on each day over the week. Of those enrolled, 37 participants provided valid accelerometer data.

An observational, environmental audit was performed in the workplace environment using an adapted version of the Checklist for Health Promotion Environments at the Worksite (CHEW) tool [30]. The CHEW instrument is designed to assess three major environments at a worksite, the physical building environment, the information environment, and the surrounding neighbourhood environment. For full details of the scoring protocol, please refer to Additional file 2. Given that CHEW was developed for use in the United States and Australia, some adaptations were made to account for the local context in Singapore [Additional file 2]. Specific elements related to SB, such as adjustable workstations and standing meeting rooms, were also assessed. A full description of the adaptation and additional elements can be found in Additional file 2. The observational audit was conducted in August 2014; both buildings of the worksite were assessed on the same day.

#### Qualitative

A focus group discussion (FGD) grounded in the socio-ecological model was conducted to elicit an in-depth understanding of the perceived individual, interpersonal, organizational and environmental determinants and identify potential strategies towards reducing SB among the participants. Topics and domains covered in the FGD included perceived difference between SB and PA, barriers to reducing SB, possible opportunities and strategies within their workplace to reduce SB. The FGD was conducted in August 2014 during working hours with six participants who met all eligibility criteria and were already enrolled into the study.

#### Analyses

All quantitative individual data (i.e., from survey, measured anthropometrics, accelerometer time sheet-working hours from workdays) were compiled into a single database. Accelerometry data processing and analysis was conducted based on the “accelerometry” package in R (Version 3.1.3) using the following established counts per minute cut points applied to the vertical axis: sedentary (<150), light (150–2019), moderate (2020–5998), vigorous (>5999) [31–33]. Only participants who wore the accelerometer for at least four days with at least 10 h per day were included in the accelerometry analyses. To account for differences in individual wear time, the proportion of time spent in each activity category relative to the entire wear time was used. CHEW data was entered and analysed descriptively separately. Using Statistical Package for the Social Science (SPSS) 23.0, descriptive analyses were conducted on individual-level data including frequencies and medians (IQR) to describe SB among the sample participants. Differences in distribution of activity minutes between workdays and non-workdays were tested using the Mann–Whitney U nonparametric test.

The focus group discussion was transcribed verbatim and thematic analysis was conducted. A priori themes were established based on the socio-ecological model for levels of influence. Two independent coders coded the data into meaning units with 80 % agreement. For each unit of disagreement, the coders held a discussion until consensus was reached. Meaning units were clustered into higher order themes.

## Results

The participants were mostly of Chinese (*n* = 33, 82.5 %) ethnicity. Majority of participants were females (*n* = 24, 60.0 %), and of the sample, nearly three-quarters had a university degree (*n* = 29, 72.5 %) (Table 1). Average body mass index was 25.0 ± 5.0 kg/m^2^ with half (*n* = 20, 50 %) the sample population categorized as overweight or obese. A quarter (*n* = 10, 25 %) of the sample reported having high cholesterol, with a few (*n* = 5, 13 %) also reporting hypertension. Across the sample, the majority (*n* = 35, 88 %) worked in building 1, while the remaining five participants worked in building 2. Other sociodemographic and health characteristics of the sample population are reported in Table 1.

**Table 1:** Sociodemographic and health characteristics of sampled participants (*n* = 40)

	n (%)
Mean ± SD age in years	43 ± 9
Ethnicity	
Chinese	33 (82.5)
Malay	2 (5.0)
Indian	3 (7.5)
Other	2 (5.0)
Gender	
Male	16 (40)
Female	24 (60)
Marital Status	
Single	13 (32.5)
Married	25 (62.5)
Divorced	2 (5)
Highest Education	
< University degree	11 (27.5)
≥ University degree	29 (72.5)
Body Mass Index^a^ in kg/m^2^:	
Mean ± SD	25.0 ± 5.0
Underweight (<18.5)	2 (5.0)
Normal (18.5–22.9)	18 (45.0)
Overweight (23–27.4)	10 (25.0)
Obese (≥27.5)	10 (25.0)
Waist Circumference mean ± SD in cm:	
Male	92.1 ± 11.2
Female	78.3 ± 10.3
Waist-to-Hip Ratio^a^:	
Male: <0.9, Female:<0.8	15 (37.5)
Male: ≥0.9, Female: ≥0.8	25 (62.5)
Self-reported health status	
High cholesterol	10 (25)
Hypertension	5 (13)
Diabetes	0 (0)

^a^cut-offs for Asian populations

### Description of sedentary behaviours

#### Accelerometer-measured

**Fig. 1:**
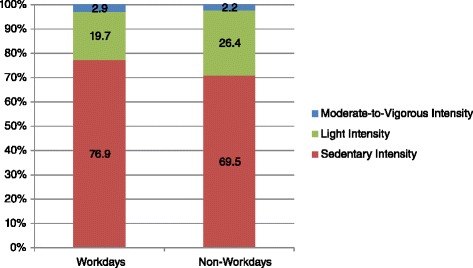
Accelerometer measured percentage of wear time by activity intensity for workdays and non-workdays

**Fig 2:**
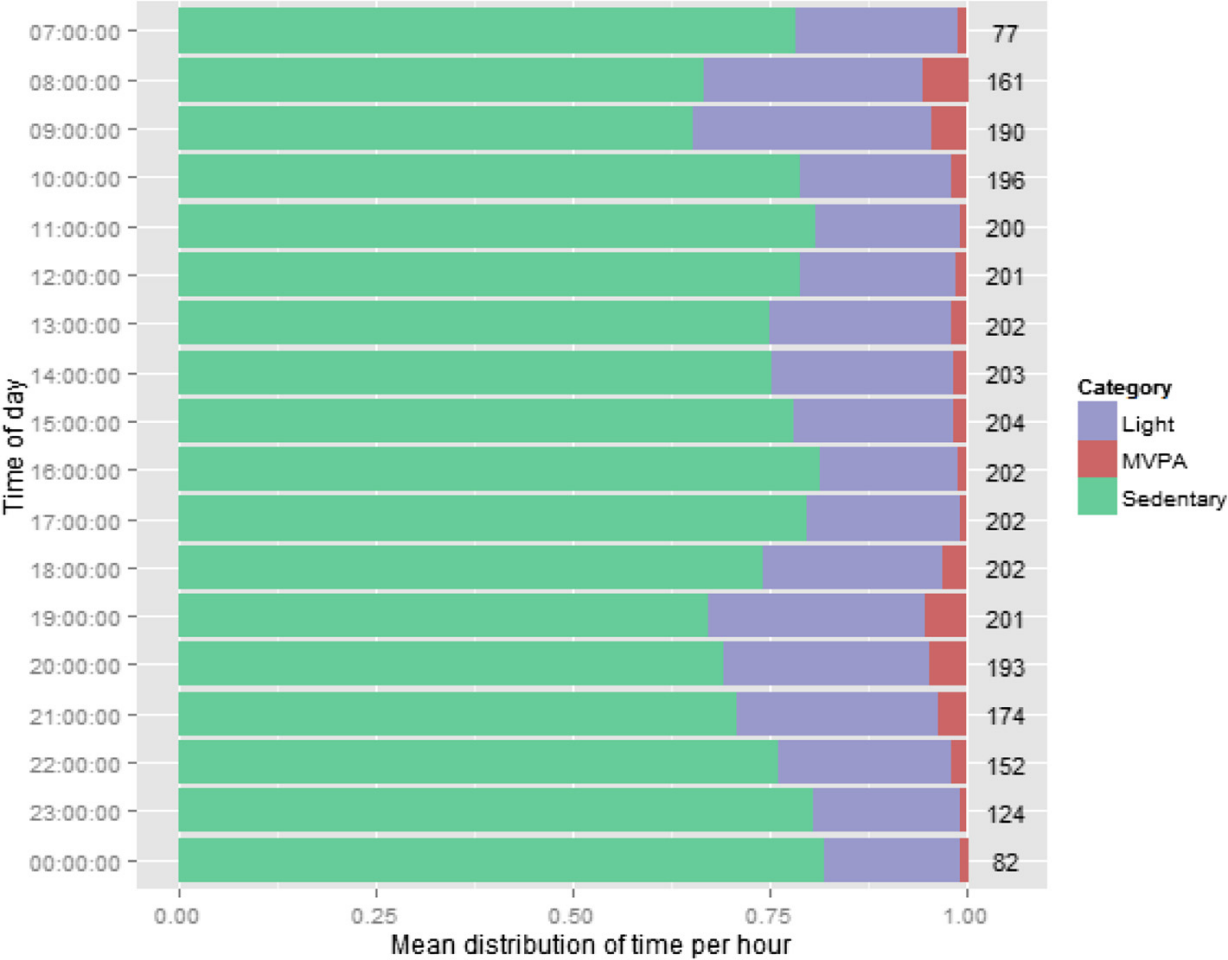
Proportion of daily hour spent in each activity category for workdays

Table 2 summarises the findings from the accelerometer assessment. Participants wore the accelerometer for a median of 7.0(2.0) days and median total wear time per day was about 14 h (856.5 min). The majority of the time was spent sedentary with more time spent during workdays (76.9 %) than on non-workdays (69.5 %; *p* < 0.01). Fig. 1 visually presents the amount of time participants spend by category of intensity by working or non-working days. Participants engaged in more light-intensity activities on non-workdays (26.4 %) than on workdays (19.7 %; *p* < 0.01). Fig. 2 summarize the time spent in different activity levels by each waking our hour throughout working days. During working hours between 1000 h and 17000 h, there is a higher percentage of time spent sedentary than other hours of the day (Fig. 2). Time spent in moderate-intensity activities was slightly higher on workdays compared to non-workdays (2.9 % vs. 2.2 %; *p* < 0.04) and the number of steps taken was similar between weekdays (7494.0) and non-weekdays (7427.0, *p* = 0.76).

**Table 2:** Accelerometer-measured activity of sampled participants (*n* = 40)^a^

	Workdays		Non-Workdays		*p*-value
	Median (IQR)		Median (IQR)		
Wear days	5.0 (1.0)		2.0 (0.0)		
Wear time/day (minutes)	875.0 (173.0)		790.0 (154.0)		<0.01*
Steps taken	7494.0 (4415.5)		7427.0 (5428)		0.76
Wear time spent/day (minutes)		%		%	
Sedentary intensity (<150 CPM)^b^	667.0 (174.5)	76.9	557.0 (158.0)	69.5	<0.01*
Light intensity (150–2019 CPM)	177.0 (79.0)	19.7	214.0 (107)	26.4	<0.01*
Moderate intensity (2020–5999 CPM)	26.0 (29.5)	2.9	19.0 (33.0)	2.2	0.04*
Moderate-to-vigorous intensity (≥2020 CPM)	27.0 (31.0)	3.0	21.0 (39.0)	2.8	0.14

^a^participants (*n* = 37) with valid wear time (at least 4 days with at least 10h per day)

^b^counts per minute (CPM) based on previously established cut points for activity intensity

*p < 0.05 between workdays and non-workdays

#### Self-reported

**Fig 3:**
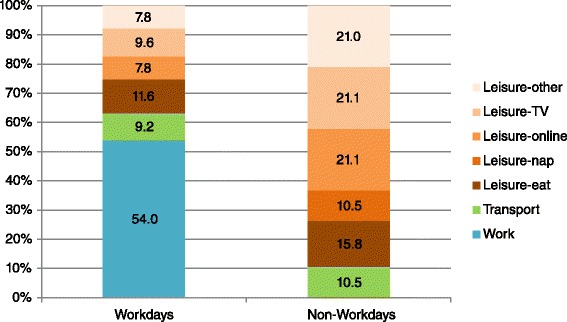
Self-reported sedentary behaviour percentage by domains for workdays and non-workdays

Self-reported total SB appears to be higher than objectively measured total SB, but patterns of SB across days of the week are similar (Table 3). The typical work week consisted of five working days for about 9.5(0.5) hours per day. Median sitting time at work was 420(180) minutes with 28 participants (70 %) reporting ≥360 min of occupational sitting time. The majority of participants (*n* = 23, 57.5 %) reported only taking 0-1 breaks per hour. Self-reported SB (e.g., sitting time) at work accounted for 54.0 % of all SB on a typical workday (Table 3). By domains, work-related SB was highest on workdays while leisure-related SB is highest on non-workdays (Fig. 3).

**Table 3:** Self-reported sedentary behavior (SB) of sampled participants (*n* = 40)

	Workdays		Non-workdays		*p*-value
Minutes of SB by domains	Median (IQR)	%	Median (IQR)	%	
work	420.0 (180.0)	54.0	-	-	-
transport	72.5 (60.0)	9.2	60.0 (90.0)	10.5	0.47
total leisure	330.0 (300.0)	36.8	585.0 (355.0)	89.5	<0.01*
eating	90.0 (60.0)	11.6	90.0 (60.0)	15.8	0.58
napping	0.0 (0.0)	0.0	60.0 (120.0)	10.5	<0.01*
online	60.0 (71.2)	7.8	120.0 (180.0)	21.1	0.03*
tv	75.0 (60.0)	9.6	120.0 (112.5)	21.1	<0.01*
other leisure	60.0 (115.0)	7.8	120.0 (120.0)	21.0	<0.01*
Minutes of total SB	870.0 (338.8)	100	650.0 (365.0)	100	<0.01*

*p < 0.5 between workdays and non-workdays

### Focus group discussion

One FGD was conducted with six participants, which included two team leaders who were heads of their departments and four team members. The demographic characteristics of FGD participants were similar to the overall sample. Of the six FGD participants, three (50 %) were of Chinese ethnicity, four (67 %) were females, and four (67 %) a university degree or higher.

In general, the participants did not have a clear conceptualization and understanding of SB and often misperceived SB as being physically inactive.

*“[Sedentary behaviour] means sitting around all day. Doing very little exercise, not moving much.”* – Team Leader

*“Well, it’s all about your approach, I guess. How you consciously move towards fitness. So, physical activity for me, at least, is being fit.”* – Team Member

However, there was strong consensus that prolonged and high prevalence of SB has negative and detrimental health consequences.

Table 4 summarises the main findings from the FGD. Factors at various levels of the socio-ecological model were identified, including the major themes of barriers and strategies to reducing SB at the workplace. At the intrapersonal level, personal factors and job scope may greatly influence an individual’s choice to be sedentary during working hours. Many of the participants commented on how the type of work they perform requires them to be at a desk with a computer. Further, participants also recognized that personal habits, such as sitting at a desk or using the lift, play an important role of occupational SB. From the interpersonal and organizational levels, social and work cultures were identified as major themes. Though it may be of personal habit, it is also very common to use the lift instead of the stairs, especially when travelling with co-workers. Participants noted that in the “Asian culture” standing during work and for meetings may not be perceived the same way as in Western culture.

**Table 4:** Barriers and strategies at reducing sedentary behaviors at the workplace

Domain	Barriers	Quotes	Strategies	Quotes
Intrapersonal				
Personal factors	• Sitting as a habit • Lack of knowledge and awareness • Lack of motivation	*“I think it’s not complicated, because, how many of us would actually climb up the stairs…”*	• Set reminders and cues to take breaks • Raise knowledge and awareness of SB and detrimental health effects	*“Target at awareness actually, rather than pushing people to do it themselves”* *“You start encouraging yourself; you also encourage other people as well.”*
Job Scope	• requires sitting • lack of time for activities • work-related stress	*“…I have this…dilemma… not being sedentary, and yet the work requires, at least from the administrative stand point, for you to be at your desk”* *“my role it’s definitely a lot of computer screen and being online monitoring”*		
Interpersonal				
Social and work culture	• Standing disturbs others • Standing is perceived as punishment or aggressive • Common to use lifts	*“doing away with emails and standing during meetings, not something that can be put into practice.”* *“…seems as though, you all stand up. Let me grill you, any more questions, out the door and then get back to work…”* *“Everybody uses the lift even if it’s one floor.”*	• Senior management and team “champions” should be role models to shift the culture around SB and PA at the workplace	*“….should come from the [head of department] as well no matter how much you spread the culture, you kind of give guidance and directions.”* *“…I have seen two [people]…he actually promote using the steps and he does it himself. I take a lot of encouragement from that”*
Organizational				
Social and work culture	• Non-personal methods of communications (e.g., emails)	*“…technology has not done any favours bringing us up in terms of the activity scale.”* *“…people who message each other even if they are in the next cubical”*	• To build capacity and social support • Providing programs and activities that encourage movement and SB reduction (e.g., incentives, standing breaks)	*“be a point not email, but to actually walk over and talk to each other…”*
Policy/Environmental				
Office Environment	• small/enclosed cubicle spaces • high availability and dispersion of printers and chairs • lack of adjustable workstations	*“The point about how we evolve…I feel that the offices are more not open floor offices”*	• Providing adjustable workstations • To have more open office floor plan that facilitates movement • Centralizing printers and other office resources	*“have digital cues…because we are working on the workstation, something like a pop out, says: ‘Did you take the stairs before your meeting?’ and you suddenly… oh, I’ll do that perhaps in my regimen next week.”*
Building Environment	• Too many lifts • Poor stair accessibility • complicated building structure	*“..sometimes you are running late for meetings, and the tendency all is just, you know, to take the lift and arrive here in a corner anyway, it’s convenient.”* *“I take lifts to second floor and I don’t know how to go beyond second floor on stairs, maybe it’s just me…”*	• To improved the accessibility of stairs • Need to improved signage to encourage stair use	*“maybe a bit more visual cues would be good as well, like, maybe at the lift landing, we just put: ‘Take the steps instead of something’ and point to a particular direction…”*

*“[Standing] will be perceived as being aggressive, very domineering!”* – Team Leader

At the policy and environmental level, participants noted the office infrastructure was hindering movement and the design could be improved. All agreed that lift usage was easier and more convenient than stairs, but also identifying the poor accessibility of stairways. Further, while novel approaches and ideas around adjustable workstations and standing or walking meetings were suggested as potential strategies for reducing SB, this would require significant intervention on the interpersonal-, organizational-, and policy- levels, including building capacity to sustain such behaviour change within the workplace.

### Environmental audit

Two buildings were included in this worksite and thus, assessed using CHEW. Table 5 presents results from the CHEW assessments, including other SB-reducing environmental items. Though the nutrition subscale of the physical environment was similar in both buildings, the physical activity subscale varied greatly. In Building 1 there were three lifts that serviced eight floors and two stairwells, while Building 2 had only one lift but nine different stairwells across four floors. Stairwells in both buildings were unlocked but not easily accessible or convenient. Aesthetic appeal of the stairwells in both buildings was poor. Passageway and hallways were adequate in width (i.e., wide enough for 2-3 people standing shoulder to shoulder), but rarely intersect each other. Lighting in the passageway of one building was dimmer due to energy-saving mechanism currently emplaced. Of the total 24 bulletin boards (12 boards in each building), only three posters were found related to specific PA behaviours (e.g., a charity run and a bicycle riding group), but none were aimed at reducing SB or encouraging overall health and wellness of office employees.

**Table 5:** Physical and informational characteristics from the observational environmental audits

	Building 1		Building 2	
	N available	Index Score	N available	Index Score
Physical Environment from CHEW^a^				
Physical activity subscale				
Showers/change rooms	5		1	
Lifts	3		4	
Stairwells	2		9	
Bicycle racks	8		0	
Subscale score		9		3
Nutrition subscale				
Vending machines	2		8	
Cafeterias/canteens	1		1	
Staff Pantries	10		6	
Subscale score		13		13.4
Physical Environment Score		22		16.4
Information Environment from CHEW				
General bulletin or information boards	12		12	
Overall health promotion signs	0		0	
Physical activity signs	0		3	
Information Environment Score		0		3
Overall CHEW score based on physical and informational environments		22		19.4
Other environmental items				
Adjustable/moving workstations	0		0	
Standing meeting rooms/common areas	0		0	
Informational environment regarding sedentary behaviors	0		0	

^a^Checklist for Health Promotion Environments at the Worksite

Of the additional elements specific to SB, none were present in the two buildings assessed. All common areas and meeting rooms had tables and chairs meant for sitting. Among the cubicle workspace, no adjustable or moving workstations were present and no supportive informational messages were identified.

## Discussion

We conducted this study to implement a comprehensive mixed-methods approach to assess SB and to better understand barriers and potential strategies towards reducing SB among office-working adults in Singapore. Our study highlights a high prevalence of SB among office workers in the sample. On a typical working day, 77 % of the time is spent in sedentary activities as objectively measured by accelerometers. This corresponds to approximately 11 h of sedentary activity during waking hours. On non-working days, recorded sedentary time is only slightly lower at 70 % of total wear time, or approximately 10 h. Completed participant questionnaire confirms these accelerometer findings as over 75 % of working hours were reportedly spent sitting.

The qualitative research component identified multiple barriers towards the reduction of SB among office workers, with particular emphasis on workplace culture and norms and the physical environment. While potential strategies were discussed, buy-in from senior management and championing employees to lead a cultural shift in workplace norms appear to be imperative. Physical office and building infrastructure are further important barriers to reducing workplace SB as lifts were easier to reach and location of stairwells were not easily found.

Results from the environmental audit confirm office layout and building infrastructure may not be conducive or help promote movement in the workplace. It is clear that the two buildings of this worksite were very different in their design and infrastructure. Though overall CHEW scores for both buildings did not differ greatly from each other, key items for the promotion of health behaviours and the subscale scores are more evident of the building differences. Additional resources, such as adjustable workstations or standing meetings/common rooms, were not available to employees. The lack of these resources was noted as barriers to reducing SB in the FGD, but participants had concerns over their acceptability within the workplace culture.

Though this is a small study, the findings are consistent with other previously published studies. Across the US, accelerometer-measured SB was near 8h per day [19]. In Australian and UK office-based workers, occupational sitting time accounted for more than half of total daily sedentary time [20, 21, 23, 24]. Though occupational sitting is heavily dependent on one’s job scope, it is clear from the present and previously published research that high levels of SB are prevalent across various populations and occupations [21, 23].

With evidence that prolonged sitting has detrimental health consequences independent of physical activity, efforts should be made to address this public health concern [9–11, 17, 23]. For these reasons, interventions have been developed and implemented to reduce workplace SB by increasing the number of breaks between bouts of sitting and by reducing overall sitting time at work [34–38]. Evidence from a recent meta-analysis supports the effectiveness of such interventions [39]. However, it was also found that the effectiveness in reducing SB appears to depend on the type of intervention strategy implemented (educational/behavioural, environmental or multi-component) and that multi-component strategies may be most effective to reduce occupational sitting [8, 37]. These findings are consistent with those in the present study.

Any effective multilevel intervention should also consider the complex cultural and social context, as well as the environmental context. A recent review of behavioural interventions to reduce SB, with some interventions conducted at worksites, conclude that the most promising technique involve some environmental modification, as well as, individual education [40]. Results from the FGD concur with a previous qualitative study, presenting similar themes, barriers and strategies for reducing occupation SB among employees [41]. In other published studies that use the CHEW or a modified version, individual items and subscale score were more often associated with behavioural outcomes than the overall composite scores [42–44]. Studies that have used CHEW often look at increasing physical activity as the behavioural outcome, but have not examined components of the physical environment for reducing SB [30, 42, 43]. Therefore, our study contributes to the existing literature by incorporating these elements to the environmental assessment.

As with all research, there are some limitations to this study. With a small sample of office-based workers in a University setting, this may not be representative of the Singapore population. However, the purpose was not to be representative but rather to develop and implement a comprehensive assessment approach and to describe SB in this population. Actigraph accelerometers were used to objectively assess SB. Although there is some debate about the most appropriate device, there is no current standard in SB assessment and using self-report questionnaires in addition to accelerometers can provide a better understanding of SB [5, 7, 15]. Further, only one FGD was conducted among the participants. While having a second FGD would have been preferred, low overall participant enrolment made recruitment for an extra FGD impossible. Results reported here are similar to other published FGD findings on acceptability and feasibility of potential strategies to reduce sedentary time among office employee [41]. Despite these limitations, results from the FGD and environmental audit provide invaluable insight when developing future intervention. Moreover, the objective of this research was to comprehensively assess SB using various methodologies, which have not been previously conducted together. The novelty of this combination approach adds to the current literature on occupational SB.

## Conclusions

Occupational sitting time in Singapore for this sample appears to be higher than, or at least as high as, in other developed nations. While a larger, representative sample of the Singaporean population is needed to confirm our findings, it seems that the factors influencing occupational SB span across the multiple levels of the socio-ecological model. The implementation of multi-level approaches may therefore help to increase the effectiveness of existing approaches and thereby contribute towards considerable population health benefits.

### Consent for publication

Not applicable.

### Availability of data

Due to restrictions set by the National University of Singapore Institutional Review Board, data are available upon request by contacting the corresponding author.

## Additional files

Additional file 1: Qualtrics Online Survey. (PDF 336 kb) Additional file 2: Detailed information for CHEW. (DOCX 15 kb)

## Abbreviations

BMI: body mass index
CHEW: checklist of health promotion environments at worksites
FGD: focus group discussion
PA: physical activity
SB: sedentary behaviour
US: United States
WHR: waist-hip ratio
