# Supplementary material for: Assessing and understanding sedentary behaviour in office-based working adults: a mixed-method approach
Source: BMC Public Health. 2016 Apr 27;16:360. doi: 10.1186/s12889-016-3023-z (PMC4847225; doi:10.1186/s12889-016-3023-z)
Supplement: Additional file 1: — Qualtrics Online Survey. (PDF 336 kb) [file 12889_2016_3023_MOESM1_ESM.pdf]

Online survey Questionnaire (Qualtrics)

SBBS Study

Q1.1 Instructions to participants   Thank you for participating in the SBBS (Sedentary Behaviour in Business School Employees) study. This online survey will ask you information on your personal particulars, followed by questions related to your sedentary and physical activities, health behaviours and status. All information provided by you is strictly confidential and will only be used for research purposes. This survey will take approximately 20 minutes of your time to complete.

Q1.2 PART I: PERSONAL PARTICULARS

Q1.3 Study ID Number (Please enter the 6 digit number provided to you)

sbbs ____________________________

Q1.4 Date of Birth (Please fill in as DD/MM/YYYY):

Q1.5 Gender :

- ❍ Male (1)
- ❍ Female (2)

Q1.6 Ethnic Group

- ❍ Chinese (1)
- ❍ Malay (2)
- ❍ Indian (3)
- ❍ Eurasian (4)
- ❍ Others (Please specify) (5) ____________________

Q1.7 Current marital status

- ❍ Single (1)
- ❍ Married (2)
- ❍ Widowed (3)
- ❍ Separated (4)
- ❍ Divorced (5)

Q1.8 Highest level of education attained (Please select one)

- ❍ No formal education/ Primary (1)
- ❍ PSLE (2)
- ❍ Secondary (3)
- ❍ O Level/ N Level (4)
- ❍ A Level (5)
- ❍ Polytechnic diploma (6)
- ❍ Other diploma and professional qualification (7)
- ❍ University and above (8)

Q1.9 Current Job Designation (Please state)

____

Q2.1 PART II. PHYSICAL ACTIVITY   In answering the following questions, 'vigorous-intensity activities' are activities that require hard physical effort and cause large increases in breathing or heart rate, 'moderate-intensity activities' are activities that require moderate physical effort and cause small increases in breathing or heart rate. You now need to think about your physical activity participation in three settings: · Activity at work · Travel to and from places · Recreational activities

Q2.2 Activity at work   Think first about the time you spend doing work.Think of work as the things that you have to do such as paid: office work, and unpaid work: household chores, or looking for a job or attending classes (if you are studying).

Q2.3 Does your work involve vigorous-intensity activity that causes large increases in breathing or heart rate, [like heavy lifting] for at least 10 minutes continuously?

- ❍ Yes (1)
- ❍ No (2)

If No Is Selected, Then Skip To Does your work involve moderate-intensity

Q2.4 In a typical week, on how many days do you do vigorous-intensity activities as part of your work? (Please indicate as number of days per week in the blank below)

____

Q2.5 On a typical day on which you do vigorous-intensity activities, how much time (in total) do you spend doing such work? (Please indicate in hours : minutes per day)

	Per day	
	Hours (1)	Minutes (2)
Hours : minutes (2)		

Q2.6 Does your work involve moderate-intensity activity that causes small increases in breathing or heart rate, [like mopping the floor or carrying light loads] for at least 10 minutes at a time?

- ❍ Yes (1)
- ❍ No (2)

If No Is Selected, Then Skip To Travel to and from places The next ques....

Q2.7 In a typical week, on how many days do you do moderate-intensity activities as part of your work? (Please indicate as number of days per week in the blank below)

Q2.8 On a typical day on which you do moderate-intensity activities, how much time (in total) do you spend doing such work? (Please indicate in hours : minutes per day)

	Per day	
	Hours (1)	Minutes (2)
Hours : minutes (1)		

Q2.10 Travel to and from places The next questions exclude the physical activities at work that you have already mentioned. Now, we would like to know about the usual way you travel to and from places (for example, going to work, shopping, market, or church, temple or mosque or going out for lunch).   Do you walk or use a bicycle (pedal cycle) for at least 10 minutes continuously to get to and from places?

- ❍ Yes (1)
- ❍ No (2)

If No Is Selected, Then Skip To Recreational activities The next que...

Q2.11 In a usual week, on how many days do you walk or bicycle (pedal cycle) for at least 10 minutes continuously to get to and from places?   (Please indicate as number of days per week in the blank below)

Q2.12 On a typical day when you walk or bicycle (pedal cycle) for at least 10 minutes, how much time (in total) do you spend walking or cycling? (Please indicate in hours : minutes per day)

	Per day	
	Hours (1)	Minutes (2)
Hours : minutes (1)		

Q2.14 Recreational activities The next questions exclude the work and transport activities that you have already mentioned. Now, we would like to know about sports, fitness and recreational activities (leisure) like swimming and badminton.   Do you do any vigorous-intensity sports, fitness or recreational (leisure) activities that cause large increases in breathing or heart rate such as running or football, for at least 10 minutes continuously?

- ❍ Yes (1)
- ❍ No (2)

If No Is Selected, Then Skip To Do you do any moderate-intensity spor...

Q2.15 In a typical week, on how many days do you do vigorous-intensity sports, fitness or recreational (leisure) activities? (Please indicate as number of days per week in the blank below)

Q2.16 How much time do you spend doing vigorous-intensity sports, fitness or recreational activities (leisure) on a typical day? (Please indicate in hours : minutes per day)

	Per day	
	Hours (1)	Minutes (2)
Hours : minutes (1)		

Q2.17 Do you do any moderate-intensity sports, fitness or recreational (leisure) activities that cause small increases in breathing or heart rate such as brisk walking, for at least 10 minutes continuously?

- Yes (1)
- No (2)

Answer If Do you do any moderate-intensity sports, fitness or recreational (leisure) activities that cause small increases in breathing or heart rate such as brisk walking, for at least 10 minutes continuous... Yes Is Selected

Q2.18 In a typical week, on how many days do you do moderate-intensity sports, fitness or recreational (leisure) activities? (Please indicate as number of days per week in the blank below)

Answer If Do you do any moderate-intensity sports, fitness or recreational (leisure) activities that cause small increases in breathing or heart rate such as brisk walking, for at least 10 minutes continuous... Yes Is Selected

Q2.19 How much time do you spend doing moderate-intensity sports, fitness or recreational activities (leisure) on a typical day?(Please indicate in hours : minutes per day)

	Per day	
	Hours (1)	Minutes (2)
Hours : minutes (1)		

Q3.1 PART III. SEDENTARY BEHAVIOUR The following questions are about sitting or reclining at work, at home, getting to and from places, or with friends, including time spent [sitting at a desk, sitting with friends, travelling in car, bus, train, reading, playing cards or watching television], but do not include time spent sleeping during the night.

Q3.2 Sedentary Behaviour How much time do you usually spend sitting or reclining on a typical day?(Please indicate in hours : minutes per day)

	Per day	
	Hours (1)	Minutes (2)
Hours : minutes (1)		

Q3.3 The following questions will ask more details about your sitting behaviour at work Sitting as part of work In a typical week, on how many days do you work? (Please indicate as number of days per week in the blank below)

Q3.4 How much time do you usually spend sitting as part of your job while at work or working from home?(Please indicate in hours : minutes per day)

	Per day	
	Hours (1)	Minutes (2)
Hours : minutes (1)		

Q3.5 Breaks in sitting time at work How many breaks from sitting (such as standing up, or stretching or taking a short walk) during one hour of sitting would you typically take at work?

- > 5 breaks per hour (1)
- 4 breaks per hour (2)
- 3 breaks per hour (3)
- 2 breaks per hour (4)
- 1 break per hour (5)
- None per hour (6)

Q3.6 Sitting or reclining not related to work Now, we would like to know how much time you spend sitting or reclining during activities not related to work. Please indicate the amount of time spent sitting or reclining during these activities on working and non-working days. (Please indicate in days per week, hours : minutes per day for working and non-working days in the following blanks below. If you do not engage in any of these activities during working or non-working days, indicate a "0" under days per week.)

Q3.7 Sitting during transportation a. Sitting in motorized transport to go to and from places

	Working Day	Non-Working Day
	Days per week (1)	Days per week (1)
Number of days per week (1)		

Answer If Transportation Sitting in motorized transport to go to and from places <p>Number of days per week</p> - Working Day - Days per week Is Greater Than 0 Or Transportation Sitting in

motorized transport to go to and from places <p>Number of days per week</p> - Non-Working Day - Days per week Is Greater Than 0

Q3.8 Sitting during transportation a. Sitting in motorized transport to go to and from places

	Working Day		Non-Working Day	
	Hours (1)	Minutes (2)	Hours (1)	Minutes (2)
Hours : minutes (Per day) (1)				

Q3.10 Sitting/Reclining during leisure time a. Eating meals (including breakfast, lunch and dinner)

	Working Day	Non-Working Day
	Days per week (1)	Days per week (1)
Number of days per week (1)		

Answer If Leisure Time   a. Eating meals (including breakfast, lunch and dinner) <p>Number of days per week </p> - Working Day - Days per week Is Greater Than 0 Or Leisure Time   a. Eating meals (including breakfast, lunch and dinner) <p>Number of days per week </p> - Non-Working Day - Days per week Is Greater Than 0

Q3.11 Sitting/Reclining during leisure time   a. Eating meals (including breakfast, lunch and dinner)

	Working Day		Non-Working Day	
	Hours (1)	Minutes (2)	Hours (1)	Minutes (2)
Hours : minutes (Per day) (1)				

Q3.12 Sitting/Reclining during leisure time   b. Napping

	Working Day	Non-Working Day
	Days per week (1)	Days per week (1)
Number of days per week (1)		

Answer If b. Napping <p>Number of days per week </p> - Working Day - Days per week Is Greater Than 0 Or b. Napping <p>Number of days per week </p> - Non-Working Day - Days per week Is Greater Than 0

Q3.13 Sitting/Reclining during leisure time   b. Napping

	Working Day		Non-Working Day	
	Hours (1)	Minutes (2)	Hours (1)	Minutes (2)
Hours : minutes (Per day) (1)				

Q3.15 Sitting while watching TV/Video/Other leisure activities c. Television viewing (i.e. Watching TV, DVD)

	Working Day	Non-Working Day
Number of days per week (1)	Days per week (1)	Days per week (1)

Answer If c. Television viewing (i.e. Watching TV, DVD) <p>Number of days per week </p> - Working Day - Days per week Is Greater Than 0 Or c. Television viewing (i.e. Watching TV, DVD) <p>Number of days per week </p> - Non-Working Day - Days per week Is Greater Than 0

Q3.16 Sitting while watching TV/Video/Other leisure activities c. Television viewing (i.e. Watching TV, DVD)

	Working Day		Non-Working Day	
	Hours (1)	Minutes (2)	Hours (1)	Minutes (2)
Hours : minutes (Per day) (1)				

Q3.17 Sitting while watching TV/Video/Other leisure activities d. Watching video (i.e. YouTube, online video), Internet surfing, social media, electronic games on any media device e.g. on computer, tablet or mobile phone

	Working Day	Non-Working Day
Number of days per week (1)	Days per week (1)	Days per week (1)

Answer If d. Watching video (i.e. YouTube, online video), Internet surfing, social media, electronic game... <p>Number of days per week </p> - Working Day - Days per week Is Greater Than 0 Or d. Watching video (i.e. YouTube, online video), Internet surfing, social media, electronic game... <p>Number of days per week </p> - Non-Working Day - Days per week Is Greater Than 0

Q3.18 Sitting while watching TV/Video/Other leisure activities d. Watching video (i.e. YouTube, online video), Internet surfing, social media, electronic games on any media device e.g. on computer, tablet or mobile phone

	Working Day		Non-Working Day	
	Hours (1)	Minutes (2)	Hours (1)	Minutes (2)
Hours : minutes (Per day) (1)				

Q3.19 Sitting while watching TV/Video/Other leisure activities e. Other leisure time activities such as sitting and reading or listening to music, playing cards, using telephones, socializing with friends or family (Please do NOT include the time you mentioned in the previous question)

	Working Day	Non-Working Day
	Days per week (1)	Days per week (1)
Number of days per week (1)		

Answer If e. Other leisure time activities such as sitting and reading or listening to music, playing car... <p>Number of days per week </p> - Working Day - Days per week Is Greater Than 0 Or e. Other leisure time activities such as sitting and reading or listening to music, playing car... <p>Number of days per week </p> - Non-Working Day - Days per week Is Greater Than 0

Q3.20 Sitting while watching TV/Video/Other leisure activities e. Other leisure time activities such as sitting and reading or listening to music, playing cards, using telephones, socializing with friends or family (Please do NOT include the time you mentioned in the previous question)

	Working Day		Non-Working Day	
	Hours (1)	Minutes (2)	Hours (1)	Minutes (2)
Hours : minutes (Per day) (1)				

Q4.1 The following questions will briefly ask about other health behaviours   PART IV. HEALTH BEHAVIOUR

Q4.2 Cigarette SmokingHave you ever smoked?

- ❍ Yes, I do smoke (1)
- ❍ Yes, I used to smoke but stopped completely (2)
- ❍ No, I never smoke (3)

If Yes, I do smoke Is Selected, Then Skip To Do you smoke?If Yes, I used to smoke but st... Is Selected, Then Skip To Alcohol Consumption In the past ...If No, I never smoke Is Selected, Then Skip To Alcohol Consumption In the past ...

Q4.3 Do you smoke?

- ❍ Daily (Please indicate the number of sticks you smoke per day in the blank below). (1) ____________________
- ❍ Occasionally (Please indicate the number of sticks you smoke per month in the blank below). (2) ____________________

Q4.4 Alcohol Consumption   In the past 12 months, how often would you have at least a standard drink?   1 standard drink refers to: 285ml regular beer OR 100ml wine OR 60ml fortified wine OR 30ml spirits

- ❍ Never (1)
- ❍ 5 or more days per week (2)
- ❍ 1 to 4 days per week (3)
- ❍ 1 to 3 days per month (4)
- ❍ Less than once a month (5)

Q4.5 Dietary Habits   How many main meal(s)* do you have in a day?   * Main meals refer to Breakfast, Lunch, Dinner

- ❍ One (1)
- ❍ Two (2)
- ❍ Three (3)

Q4.6 How often do you eat out?

- Never (1)
- Per month (Please indicate the number of times per month in the blank below) (2) ____________________
- Per week (Please indicate the number of times per week in the blank below) (3) ____________________

Q4.7 How many serve(s) of vegetables do you usually eat in a day? (Please indicate your answer in the blank below) 1 serve = ¾ Mug cooked OR ¼ of a plate (100g) Reference: Pictorial taken from Health Promotion Board 2012

Number of serving(s) per day (1)

Q4.8 Excluding fruit juices and dried fruits, how many serve(s) of fruits do you usually eat in a day?(Please indicate your answer in the blank below) An example of one serving: 1 wedge papaya OR 1 wedge watermelon (130g) OR 1 medium banana OR 10 grapes (50g) Reference: Pictorial taken from Health Promotion Board 2012

Number of serving(s) per day (1)

Q4.9 How many serve(s) of whole grain products do you usually eat in a day? (Please indicate your answer in the blank below) Reference: Pictorial taken from Health Promotion Board 2012

Number of serving(s) per day (1)

Q4.10 What types of fats do you use for cooking at home?

- Butter, dripping, ghee, lard (1)
- Vegetable oil, palm oil, blended oil (2)
- Corn oil, soyabean oil, sunflower oil, safflower oil (3)
- Peanut oil, canola oil, olive oil (4)
- Others (Please specify in the blank below) (5) ____________________
- Do not cook at home at all (6)

Q4.11 How often do you drink sweetened drinks*? *Examples of sweetened drinks include: Soft drinks, fruit drinks, packet drinks, cordials etc.

- Never (1)
- Per week (Please indicate the number of times per week in the blank below) (2) ____________________
- Per month (Please indicate the number of times per month in the blank below) (3) ____________________

Q5.1 Keep going! You are now at the final section of the survey. Lastly, the following questions will ask you regarding your current health status, quality of life and workplace well being. Q5.2 PART V. HEALTH, QUALITY OF LIFE AND WORKPLACE WELL BEING Health status Have you ever been told by a doctor (western trained) that you have one of the following medical conditions?

	No (1)	Yes (2)	Not sure (3)
High Cholesterol (1)	❍	❍	❍
Hypertension (2)	❍	❍	❍
Diabetes (3)	❍	❍	❍
Kidney failure (4)	❍	❍	❍
Heart failure (5)	❍	❍	❍
Heart attack (6)	❍	❍	❍
Stroke (7)	❍	❍	❍
Cancer (8)	❍	❍	❍
Other heart diseases (i.e.: angina, coronary/ischaemic heart disease, congential heart disease, cardiomyopathy, cardiomegaly) (9)	❍	❍	❍

Q5.3 **Musculoskeletal symptoms (Adapted from Kuorinka1987) Have you at any time during the last 12 months had trouble (ache, pain, discomfort)?

	No (1)	Yes (2)
Neck (1)	❍	❍
Right shoulder (2)	❍	❍
Left shoulder (3)	❍	❍
Both shoulders (4)	❍	❍
Right elbow (5)	❍	❍
Left elbow (6)	❍	❍
Both elbows (7)	❍	❍
Right wrist/hand (8)	❍	❍
Left wrist/hand (9)	❍	❍
Both wrists/hands (10)	❍	❍
Upper back (11)	❍	❍
Lower back (12)	❍	❍
One or both hips/thighs (13)	❍	❍
One or both knees (14)	❍	❍
One or both ankles/feet (15)	❍	❍

Q5.4 Answer this question if you have indicated a 'Yes' in the previous question of **Musculoskeletal symptoms. Have you at any time during the last 12 months been prevented from doing your normal work (at home or away from home) because of the cause of trouble?

	No (1)	Yes (2)
Neck (1)	❍	❍
Shoulders (2)	❍	❍
Elbows (3)	❍	❍
Wrists/Hands (4)	❍	❍
Upper back (5)	❍	❍
Lower back (6)	❍	❍
One or both hips/thighs (7)	❍	❍
One or both knees (8)	❍	❍
One or both ankles/feet (9)	❍	❍

Q5.5 Answer this question if you have indicated a 'Yes' in the previous question of **Musculoskeletal symptoms. Have you had trouble at any time during the last 7 days?

	No (1)	Yes (2)
Neck (1)	❍	❍
Shoulders (2)	❍	❍
Elbows (3)	❍	❍
Wrists/Hands (4)	❍	❍
Upper back (5)	❍	❍
Lower back (6)	❍	❍
One or both hips/thighs (7)	❍	❍
One or both knees (8)	❍	❍
One or both ankles/feet (9)	❍	❍

Q5.7 Quality of Life (Reference from SF-12, Health Survey short form 12) In general, would you say your health is:

❍ Excellent (1)
❍ Very Good (2)
❍ Good (3)
❍ Fair (4)
❍ Poor (5)

Q5.8 The following questions are about activities you might do during a typical day. Does your health now limit you in these activities? If so, by how much?

	Yes, limited a lot (1)	Yes, limited a little (2)	No, not limited at all (3)
Moderate activities, such as moving a table, pushing a vacuum cleaner, bowling or playing golf (1)	❍	❍	❍
Climbing several flights of stairs (2)	❍	❍	❍

Q5.9 During the past 4 weeks, have you had any of the following problems with your work or other regular daily activities as a result of your physical health?

	Yes (1)	No (2)
Accomplished less than you would like (1)	❍	❍
Were limited in the kind of work or other activities (2)	❍	❍

Q5.10 During the past 4 weeks, have you had any of the following problems with your work or other regular daily activities as a result of any emotional problems (such as feeling depressed or anxious)?

	Yes (1)	No (2)
Accomplished less than you would like (1)	❍	❍
Didn’t do work or other activities as carefully as usual (2)	❍	❍

Q5.11 During the past 4 weeks how much did pain interfere with your normal work (including both work outside the home and housework)?

❍ Not at all (1)
❍ A little bit (2)
❍ Moderately (3)
❍ Quite a bit (4)
❍ Extremely (5)

Q5.12 The following questions are about how you feel and how things have been with you during the past 4 weeks. For each question, please indicate one answer that comes closest to the way you have been feeling.   How much time during the last 4 weeks:

	All of the time (1)	Most of the time (2)	A good bit of the time (3)	Some of the time (4)	A little of the time (5)	None of the time (6)
Have you felt calm and peaceful? (1)	❍	❍	❍	❍	❍	❍
Did you have a lot of energy? (2)	❍	❍	❍	❍	❍	❍
Have you felt downhearted and blue? (3)	❍	❍	❍	❍	❍	❍

Q5.13 During the past 4 weeks, how much of the time has your physical health or emotional problems interfered with your social activities (like visiting friends, relatives etc)?

❍ All of the time (1)
❍ Most of the time (2)
❍ A good bit of the time (3)
❍ Some of the time (4)
❍ A little of the time (5)
❍ None of the time (6)

Q5.15 Well done! Keep up the great effort! You are almost near to completion. This is the final part of the survey.   Work place well being (Reference from Schaufeli & Bakker 2003)   The following 9 statements are about how you feel at work. Please read each statement carefully and decide if you ever feel this way about your job. If you have never had this feeling, indicate “0” after the statement. If you have had this feeling, indicate how often you felt (from 1 to 6), that best describes how frequently you feel that way.

	Never 0 Never (1)	Almost never 1 A few times a year or less (2)	Rarely 2 Once a month or less (3)	Sometimes 3 A few times a month (4)	Often 4 Once a week (5)	Very often 5 A few times a week (6)	Always 6 Everyday (7)
1. At work, I feel bursting with energy (1)	❍	❍	❍	❍	❍	❍	❍
2. At my job, I feel strong and vigorous (2)	❍	❍	❍	❍	❍	❍	❍
3. I am enthusiastic about my job (3)	❍	❍	❍	❍	❍	❍	❍
4. My job inspires me (4)	❍	❍	❍	❍	❍	❍	❍
5. When I get up in the morning, I feel like going to work (5)	❍	❍	❍	❍	❍	❍	❍
6. I feel happy when I am working intensely (6)	❍	❍	❍	❍	❍	❍	❍
7. I am proud on the work that I do (7)	❍	❍	❍	❍	❍	❍	❍
8. I am immersed in my work (8)	❍	❍	❍	❍	❍	❍	❍
9. I get carried away when I am working (9)	❍	❍	❍	❍	❍	❍	❍

Q5.16 Awesome! You have completed the survey. Thank You for your participation! Have a nice day ahead!
