# Supplementary material for: Assessing and understanding sedentary behaviour in office-based working adults: a mixed-method approach
Source: BMC Public Health. 2016 Apr 27;16:360. doi: 10.1186/s12889-016-3023-z (PMC4847225; doi:10.1186/s12889-016-3023-z)
Supplement: Additional file 2: — Detailed information for CHEW. (DOCX 15 kb) [file 12889_2016_3023_MOESM2_ESM.docx]

Scoring Protocols

Based on CHEW, the physical characteristics within the building (e.g., stairs, lifts, lighting, etc.), features of the information environment (e.g., boards and signage), and the surrounding areas (e.g., parking areas, sidewalks, cafeteria and other eating areas, etc.) were systematically assessed [30]. Each of these three broad categories is scored based on the items in their subscale. A subscale score may be based on the sum of items available or a ratio of items within the subscale. An overall score was given to each building based on the sums from the three categories [30]. Buildings assessed with CHEW can be compared on individual items, subscale scores, or overall composite index score. The CHEW tool has shown to be a valid and reliable instrument to quantify environment influences on health promotion in the workplace [30].

Adaptation to local context

For this study, the original 112-item of the CHEW tool was modified to account for the local context of the buildings and surrounding environment. For example, fitness centres and workplace gyms are not typically found in Singapore. Therefore, these original subscales of the CHEW could not be assessed according to the protocol. Further, since a large majority of Singapore’s population take public transportation, many worksites do not have designated vehicle parking areas and surrounding grounds around the workplace is often public space. Due to these contextual differences, standard operational definitions were established for Singapore. For the purpose of this study, the overall CHEW score is based on the sum of the physical environment, including the physical activity and nutrition subscales, and the information environment. Additionally, in light of the objectives to assess workplace SB and since CHEW had previously not been developed for this explicit purpose, some other environmental elements were assessed. These elements were selected based on a systematic review of office-based interventions to reduce SB and other plausible environmental approaches [17, 31]. They included, adjustable and/or moving workstations (or other forms of activity permissive workstations, such as pedal bikes), standing meeting rooms or other purposefully designed standing common areas, and informational posters/prompts with the specific message of reducing SB were among the additional elements assessed. A simple description of presence and availability were recorded for these additional elements.
